# Study on the Anticoagulant or Procoagulant Activities of Type II Phenolic Acid Derivatives

**DOI:** 10.3390/molecules22122047

**Published:** 2017-11-28

**Authors:** Xuan Luo, Chuanrong Du, Hui Cheng, Jian-hua Chen, Cuiwu Lin

**Affiliations:** 1 School of Chemistry and Chemical Engineering, Guangxi University, 100 Daxue Road, Nanning 530004, Guangxi, China; luo-xuan625@hotmail.com (X.L.); duchuanrong123@163.com (C.D.); chui240514119@sina.com (H.C.); 2 Guangxi Colleges and Universities Key Laboratory of Applied Chemistry Technology and Resource Development, Guangxi University, 100 Daxue Road, Nanning 530004, Guangxi, China; 3 School of Resources, Environment, and Materials, Guangxi University, 100 Daxue Road, Nanning 530004, Guangxi, China; jhchen@gxu.edu.cn

**Keywords:** anticoagulant, procoagulant, haemostatic, phenolic acid, surface plasmon resonance

## Abstract

In this study, three type II phenolic acids (caffeic acid, *p*-hydroxycinnamic acid, and ferulic acid) were used to synthesize a total of 18 phenolic acid derivatives. With molecular docking for molecule design and target protein (factors) screening, in combination with the confirmation of target proteins (factors) by surface plasmon resonance, and the evaluation of haemostatic and anticoagulant activities with five blood assays (plasma recalcification time, prothrombin time, activated partial thromboplastin time, fibrinogen, and thrombin time), the data indicated that caffeic acid derivatives showed certain anticoagulant or procoagulant activities and that two other series contained compounds with the best anticoagulant activities. Using Materials Studio analysis, particular functional groups that affect anticoagulant or procoagulant activities were revealed, and these conclusions can guide the discovery of compounds with better activities.

## 1. Introduction

Phenolic acid compounds, which play essential roles in growth, development, or reproduction, are a group of non-flavonoid polyphenols that exist in most plant tissues as secondary metabolites [1]. Additionally, these phenolic compounds are important components of the human diet due to their potential antioxidant activities [1,2]. With immense research on pharmacological activity, phenolic acid compounds have shown many bioactivities, not only including familiar antioxidant [3,4,5], antibacterial [6,7], and anti-inflammatory [8,9] activities, but also haemostatic [10,11], anticancer [12,13], and antiviral [14,15] activities. Due to their excellent physical and chemical properties, phenolic acid compounds have aroused much attention and become one of the hotspots in drug development.

Some studies have indicated that phenolic acid compounds not only have haemostatic activity [16], but also show better anticoagulant activity [17,18,19,20,21,22]. Since 2002, our group has focused on studying anticoagulant activity components in *Blumea riparia* DC. and has obtained some polysaccharide, flavonoid, and phenolic acid compounds [10,11,23,24,25,26,27,28]. Additionally, the obtained flavonoids and phenolic acids were used as parent compounds to synthesize a series of derivatives, and their haemostatic and anticoagulant activities were evaluated by several bioassays [29,30,31,32,33,34,35,36,37,38]. According to our previous research results, phenolic acid compounds separated from *Blumea riparia* DC. have good haemostatic and anticoagulant activities and could be precursors to developing new haemostatic and anticoagulant drugs.

According to their structural characteristics, all phenolic acid compounds have a carboxyl group attached or linked to a benzene ring [39], and these compounds can be classed into two types: type I, benzoic acid derivatives, and type II, cinnamic acid derivatives (hydroxycinnamic acids) [40]. For Type II, caffeic, *p*-hydroxycinnamic, ferulic, and sinapic acids are common. Notably, the structures of most haemostatic drugs used in the clinic include phenolic hydroxyl, amide, amino, and carboxyl groups. In the present study, based on this information, three Type II phenolic acids (caffeic acid, *p*-hydroxycinnamic acid, and ferulic acid) were used as precursors to synthesize a series of derivatives. Then, the haemostatic and anticoagulant activities of these derivatives were evaluated to guide the design of new haemostatic and anticoagulant drugs.

## 2. Materials and Methods

### 2.1. Apparatus and Software

Melting points were measured on an X-4 microscopic melting point apparatus (Beijing Tech Instruments, Beijing, China). Ultraviolet (UV) spectra were recorded on a TU-1900 UV/VIS spectrophotometer (Shanghai Jinghua Technology Instruments, Shanghai, China). Fluorescence data were recorded on a Cary Eclipse fluorescence spectrophotometer (Agilent Technologies, Santa Clara, CA, USA). Infrared (IR) spectra were determined using a Nicolet NEXUS470 spectrophotometer with KBr pellets (Thermo Fisher Scientific, Waltham, MA, USA). Fast atom bombardment mass spectrometry (FAB-MS) data were determined by a VGZAB-HS mass spectrometer (Thermo Fisher Scientific, Waltham, MA, USA). Surface plasmon resonance (SPR) measurements were performed on a ProteOn XPR36 Protein Interaction Array system (Bio-Rad Laboratories, Hercules, CA, USA) using a GLH chip. Nuclear magnetic resonance (NMR) spectra were recorded on an Advance III HD 600 MHz spectrometer (Bruker, Zurich, Swiss) with tetramethylsilane (TMS) as an internal standard for deuterated organic solvents or 2,2,3,3-*d*_4_-3-(trimethylsilyl) propionic acid sodium salt (TSP) as an internal standard for deuterium oxide. Prothrombin time (PT), activated partial thromboplastin time (APTT), fibrinogen (FIB), and thrombin time (TT) were recorded on an LG-PABER-I semi-automated coagulation analyser (Steellex Scientific Instrument, Beijing, China). A Milli-Q Advantage A10 (Merck Millipore, Billerica, MA, USA) supplied the pure water used in these experiments.

The molecular structures were drawn by Materials Studio (Accelrys Software, BIOVIA, San Diego, CA, USA) to study the distances of some atoms in the structures.

### 2.2. Reagents

Three material phenolic acids (caffeic acid, *p*-hydroxycinnamic acid, and ferulic acid) and *p*-aminomethylbenzoic acid (*p*-AMBA), the positive control for PT, APTT, FIB, and TT, were purchased from Zhuhai Jiaxinkang Pharmaceutical Technology Co. (Zhuhai, China). Fibrinogen was purchased from Beijing Qinyuanhuzhi Biotechnology Co. (Beijing, China). Thrombin and deuterated solvents for NMR analysis were purchased from Sigma-Aldrich Corp. (St. Louis, MO, USA). APTT, PT, TT, and FIB kits were supplied by HEALL Bio-Science Technology (Qingdao) Co. (Qingdao, Shandong, China). Other chemicals and reagents were supplied by Shanghai Aladdin Biochemical Technology Co. (Shanghai, China), Guangdong Guanghua Sci-Tech Co. (Shantou, Guangdong, China), and Xilong Scientific Co. (Shantou, Guangdong, China).

Healthy human plasma (the platelet-poor plasma, PPP) was supplied by The First Affiliated Hospital of Guangxi Medical University (Nanning, Guangxi, China), and all five blood assays were conducted in accordance with the Declaration of Helsinki. The protocol was approved by the Ethics Committee of First Affiliated Hospital of Guangxi Medical University (Project identification code: 2017(KY-E-050)).

### 2.3. Preparation of Phenolic Acid Derivatives

#### 2.3.1. Caffeic Acid Derivatives

Preparation of diacetyl caffeic acid: Caffeic acid (50 g), 600 mL acetic anhydride and pyridine were added to a 1000 mL round-bottom flask and reacted at room temperature for 12 h with stirring. Subsequently, the mixture was transferred into a 5 L beaker and eight-fold iced water was added under violent stirring. The mixture was left standing for 24 h and yielded a white precipitate. After vacuum filtration, washing, and 60 °C oven drying, diacetyl caffeic acid was obtained and ready for the next reaction without further purification.

**Scheme 1:**
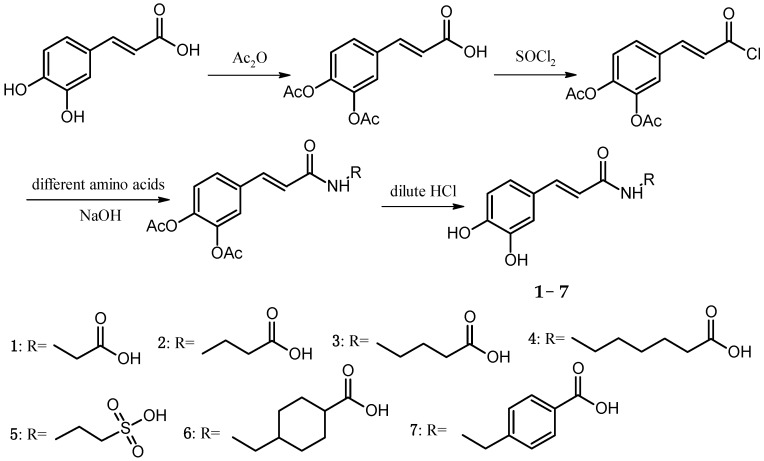
Synthetic route of Cds. **1**–**7**.

Preparation of Cds **1**–**7**: Diacetyl caffeic acid (20 mmol) and 15 mL SOCl_2_ were added into a 50 mL round-bottom flask and reacted in a 60 °C oil bath for 5 h with stirring. Subsequently, the solvents were removed by a rotary evaporator, and diacetyl caffeic acyl chloride was obtained. Glycine, β-alanine, γ-propalanine, 6-amidocaproic acid, taurine, tranexamic acid, or *p*-aminomethylbenzoic acid (40 mmol) was dissolved in 20 mL of a 2 M NaOH solution in a 50 mL round-bottom flask. At 10–15 °C in a water bath with stirring, diacetyl caffeic acyl chloride THF solution (adding 5 mL THF to dissolve obtained diacetyl caffeic acyl chloride) was slowly added to the flask. After a 2.5 h reaction, the pH of the mixture was adjusted to **4**–**5** by adding diluted HCl. The mixture was left standing at room temperature for 24 h, followed by vacuum filtration, washing, and 60 °C oven drying to obtain Cds. **1**–**7** (The Synthetic route of Cds. **1**–**7** was shown in Scheme 1).

The melting points of Cds. **1**–**7** were measured, and their structures were confirmed by using a battery of spectroscopic methods, including IR, ESI-MS, and NMR. The yields, appearances, melting points, and ESI-MS data of Cds. **1**–**7** were listed in Table 1 and the IR data were listed in Table 2.

#### 2.3.2. *p*-Hydroxycinnamic acid Derivatives

**Scheme 2:**
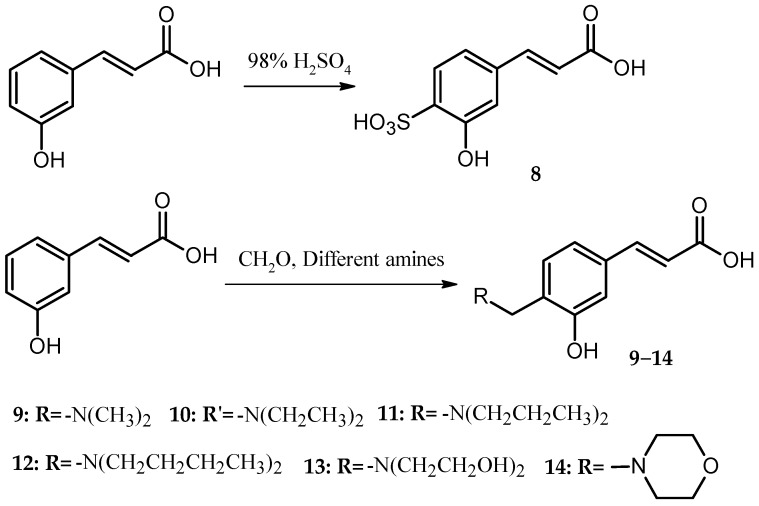
Synthetic Route of Cds. **8**–**14**.

Preparation of Cd. **8**: *p*-hydroxycinnamic acid (20 mmol) and 30 mL of concentrated H_2_SO_4_ were added to a 100 mL round-bottom flask and reacted for 8 h in a 60 °C oil bath with stirring. Subsequently, the mixture was transferred into a 250 mL beaker, and 10 mL of distilled water was added (too viscous to handle in the next step). With stirring, solid NaOH was added to the beaker until no more precipitate was yielded. After vacuum filtration, washing with distilled water, and recrystallization in distilled water, Cd. **8** was obtained, and the yield was 45.1% (The Synthetic route of Cds. **9**–**14** was shown in Scheme 2).

Preparation of Cds. **9**–**14**: *p*-Hydroxycinnamic acid (37 mmol), 50 mL methanol and formaldehyde–water solution (36%, containing 49 mmol formaldehyde) was added into a 250 mL round-bottom flask and reacted for 8 h in a 60 °C oil bath with reflux and stirring. After the mixture appeared clean, 104 mmol dimethylamine, diethylamine, di-*n*-propylamine, di-*n*-butylamine, diethanolamine, or morpholine was added to the flask, and the reaction was continued for 5 h. Large quantities of solid were precipitated in the flask. After vacuum filtration, washing with methanol, and 60 °C oven drying, Cds. **9**–**14** were obtained.

The melting points of Cds. **9**–**14** were measured, and their structures were confirmed by using a battery of spectroscopic methods, including IR, ESI-MS, and NMR. The yields, appearances, melting points, and ESI-MS data of Cds. **9**–**14** were listed in Table 3 and the IR data were listed in Table 4.

#### 2.3.3. Ferulic Acid Derivatives

**Scheme 3:**
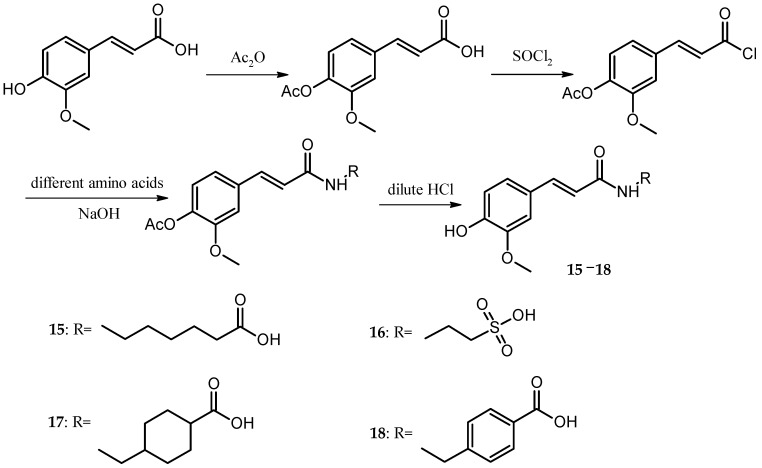
Synthetic route of Cds. **15**–**18**.

The preparation of Cds. **15**–**18** was similar to the preparation of Cd. **1**. The only differences were that the amount of acetic anhydride was reduced to half in the preparation of acetyl ferulic acid, and the amino acids were changed to 6-amidocaproic acid, taurine, tranexamic acid, or *p*-aminomethylbenzoic acid (The synthetic route of Cds. **15**–**18** was shown in Scheme 3).

The melting points of Cds. **15**–**18** were measured, and their structures were confirmed by using a battery of spectroscopic methods, including IR, ESI-MS, and NMR. The yields, appearances, melting points, and ESI-MS data of Cds. **15**–**18** were listed in Table 5 and the IR data were listed in Table 6.

### 2.4. Structure Characterization

^1^H- and ^13^C-NMR data assignments of some compounds were achieved by DEPT and 2D NMR (COSY, HSQC, and HMBC). All NMR data are provided in the supplementary information.

### 2.5. Molecular Docking

#### 2.5.1. Virtual Screening

The targets of the phenolic acid derivatives (PADs) were searched by PharmMapper Server, a web-based tool designed to identify potential target candidates for the given small molecules via ‘reverse’ pharmacophore mapping [41]. The optimized structures of PADs were prepared by ACD/ChemSketch (Version 12.6, Advanced Chemistry Development, Inc., Toronto, ON, Canada) and Open Babel [42] (Version 2.3.2, http://openbabel.org; accessed Oct 2014) in the mol2 format and submitted to PharmMapper (http://59.78.96.61/pharmmapper/; access date: 12 September 2012) for the prediction of proteins with three-dimensional structures and PAD binding sites in the Protein Databank. In the present study, the number of reserved matched targets is defined as 300.

#### 2.5.2. AutoDock Docking

AutoDock is one of the most frequently used molecular docking suites and has been extensively validated over the years [43,44]. Calculations were performed using AutoDock 4.2.1.5, which is an automated docking suite capable of performing rigid or flexible docking [45]. The following program settings were used in the present study: 100 runs with 2,500,000 energy evaluations and a maximum number of 27,000 generations.

### 2.6. Surface Plasmon Resonance

Surface plasmon resonance (SPR) biosensors are optical sensors exploiting special electromagnetic waves, surface plasmon polaritons, to probe interactions between an analyte in solution and a biomolecular recognition element immobilized on the SPR sensor surface [46]. In a typical experiment, fibrinogen and thrombin were immobilized (approximately 12,000 RU) in flow cells, with one flow cell used as a blank. For the screening experiment, the ligands were diluted to 10 μM with running buffer (12 mM HEPES, 4 mM Tris, 1 mM EDTA, 1.5 mM MgCl_2_ and 0.005% Tween-20). The PAD solutions were diluted to different concentrations with running buffer. The ligand was injected at a flow rate of 25 μL/min for 180 s during the association phase, followed by a 300 s dissociation phase at 25 °C. The GLH chip was regenerated with a short injection of 1 M NaCl between consecutive measurements. The final graphs were obtained by subtracting blank sensorgrams from the sensorgrams for fibrinogen or thrombin.

Kinetic and equilibrium analyses were performed using the ProteOn manager software. The theoretical max RU of the compounds was evaluated via the following equation:

(1) $$R_{\max}=R_{\mathrm{protein}}r_{\mathrm{protein}/\mathrm{ligand}}(MW_{\mathrm{protein}}/MW_{\mathrm{ligand}})$$

where *R*_protein_ is 12,000 RU, the amount of protein immobilized on the chip; *r*_protein/ligand_ is 1:1, the ratio of the protein–ligand interaction; MW_protein_ is the molecular weight of fibrinogen or thrombin; and MW_ligand_ is the molecular weight of the compounds. The kinetic SPR sensorgrams were fitted with a 1:1 Langmuir binding mode, and the *K*_D_ values were achieved by the Analysis module in ProteOn XPR36 software. The equation of the association process is:

(2) $$d[\mathrm{AL}]/dt=k_{a}[A][L]-k_{d}[\mathrm{AL}]$$

where, in the dissociation process, [A] = 0; therefore, d[AL]/d*t* = −*k*_d_[AL]. [AL] is the concentration of the complex, *t* is time, and [A] and [L] are the concentrations of the analyte and the substance, respectively. *k*_a_ and *k*_d_ are the association and dissociation rate coefficients, respectively. *K*_D_ was calculated from *k*_a_ and *k*_d_ using the following equation:

(3) $$K_{D}=k_{d}/k_{a}$$

### 2.7. Measurement of Plasma Recalcification Time

For this analysis, 0.1 mL PPP (defrost and incubate at 37 °C) and 0.1 mL of the different sample solutions were mixed well in test tubes (8 mm diameter). After incubation for 5 min in a 37 °C water bath, 0.1 mL CaCl_2_ solution (0.025 mM) was respectively added to each tube, and the process was monitored. The period until silky fibrin appeared was recorded as the PRT. The negative control was saline, and the positive control was *p*-aminomethylbenzoic acid.

### 2.8. Measurement of APTT, PT, TT, and FIB

In a 2 mL centrifuge tube, 0.9 mL PPP and 0.1 different sample solution mixed well and incubated at 37 °C for 5 min. APTT, PT, TT, and FIB were detected using blood coagulation factor assay kits according to the manufacturer’s instructions. The negative control was saline, and the positive control was *p*-aminomethylbenzoic acid.

## 3. Results and Discussion

In vivo, haemostasis and blood circulation for removing blood stasis are two contradictory activities. However, both activities objectively exist at the same time. In gynaecological diseases or after surgery, wounds require stopping bleeding, but the medicines used cannot increase patient blood viscosity, which will form thrombosis; at the same time, the medicines are needed to slowly dissipate the congestion in blood circulation and promote the restoration of the patient’s body.

**Figure 1:**
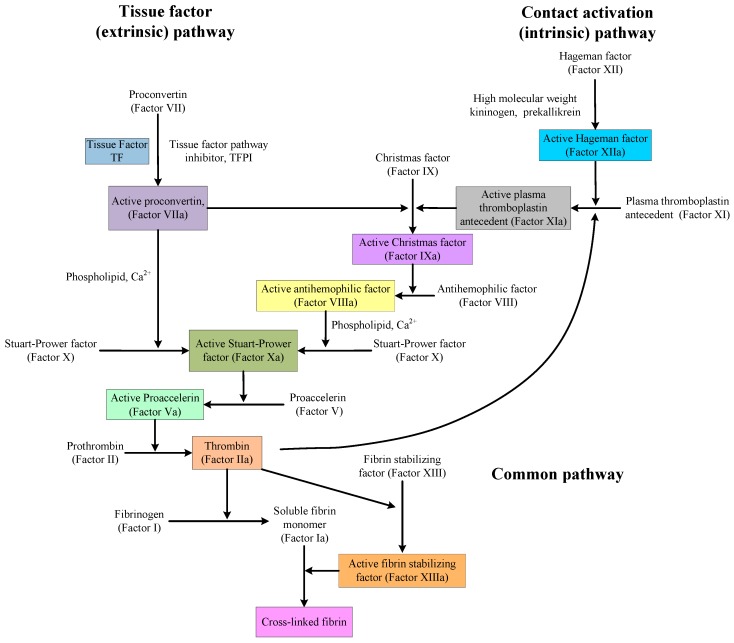
The classical blood coagulation pathway [49].

There is also a paradox in discovering and designing medicines that affect the mechanisms of anticoagulation and procoagulation. As shown in Figure 1, the coagulation pathway is very complex, but the situation is different near thrombin. Indeed, in that area, there is single factor control. Therefore, in the design of medicines or the software simulation of medicine activity, thrombin or other proteins and factors in that area are, in many cases, used as the targets. However, no matter how to design or evaluate medicines, all obtained compounds and precursors will be used in actual blood systems to evaluate their activities, and the activities of these compounds are a combination of their performance on every protein and factor in the coagulation pathway. The compounds, showing good anticoagulation activity with thrombin, may not have the same anticoagulation activity or procoagulant effect in blood.

In the present study, the anticoagulant and procoagulant activities of three phenolic acid derivative series, 18 PADs, were investigated. Comparing the results of molecular docking and SPR with those of five blood assays, we found that using molecular docking and SPR to assist the molecule design had some drawbacks (one of the reasons was the paradox mentioned above). However, the experimental data also showed that using molecular docking and SPR could generate some important information for molecule design.

### 3.1. Virtual Screening

Virtual screening of chemical libraries, as an alternative approach for the identification of new lead compounds in drug discovery, has been widely used [47]. As the structures of biomolecules deposited in the Protein Data Bank (PDB) have substantially increased in the past decades, searching for the targets of a given drug or small compound (also known as inverse screening, target fishing, off-target prediction, etc.) has become a useful approach [48]. In PharmMapper tool, 18 newly-synthesized PADs were aligned to 2241 human protein targets, among which the top 300 targets were analysed. From the results, three types, a total of seven protein targets, were screened out. The first type was targets associated with coagulation, including thrombin, prothrombin, coagulation factor X, and fibrinogen. The second type was targets associated with anticoagulant, including antithrombin-III and plasminogen. The third type was the two-way regulatory target, vitamin K-dependent protein Z.

### 3.2. AutoDock Docking

The results of the docking study for all 18 synthesized PADs with seven protein targets, including thrombin (3RLW), prothrombin (1YPK), coagulation factor X (2J4I), fibrinogen (3GHG), antithrombin-III (2ANT), plasminogen (1QRZ), and vitamin K-dependent protein Z (3H5C), are listed in Table 7.

For the caffeic acid derivative series (Cds. **1**–**7**), the binding energies of Cd. **5** with protein targets were lower than those of the other caffeic acid derivatives. Cds. **8**–**14** were *p*-hydroxycinnamic acid derivatives, and the binding energy data for some compounds revealed three important findings: The docking energies of Cd. **8** with the seven protein targets were very low (six of the seven energy values were the lowest compared with those of the other 17 compounds, and the remaining value was the second to the lowest value). In contrast, Cd. **12** had the best docking results among the 18 compounds (although the binding energy with 1QRZ was not the highest value, this value was still the second highest value). Except for Cd. **8**, all other *p*-hydroxycinnamic acid derivatives had better docking results with 3GHG and 2ANT, and binding with these two protein targets would release greater than −20 kJ∙mol^−1^ of energy. For the four ferulic acid derivatives (Cds. **15**–**18**), one derivative, Cd. **16**, showed lower binding energies with the protein targets.

Three different series of PADs, Cds. **5**, **8**, and **16**, had worse docking results and a similar structure fraction, the sulfonic acid group. These results suggest that in the docking calculation, the sulfonic acid group could affect the binding between PADs and protein targets, particularly in connection with the benzene ring in the PAD structure. Since the sulfonic acid group could withdraw electrons out of the benzene ring, the low electron density generated worse docking results for Cd. **8**.

A comparison of the average of each protein target with the 18 PADs revealed four coagulation targets with better docking results, particularly 3RLW with caffeic acid derivatives and 3GHG with *p*-hydroxycinnamic acid derivatives.

### 3.3. Surface Plasmon Resonance Results

Based on the AutoDock docking results, thrombin and fibrinogen were the protein targets used in SPR analysis.

Autodock can predict the combination of small molecules and proteins by theoretical calculation. Moreover, to obtain the results, the calculation is based on the electronic distribution of the molecule itself (such that the electron-rich regions in the electrostatic potential map of the compound will combine with the electron deficient regions of the protein) and intermolecular interactions, including van der Waals, electrostatic, and bond interactions. During the calculation, the effects of the external environment are considered. Thus, the AutoDock results were not consistent with the results of surface plasmon resonance (SPR), listed in Table 8.

In Table 8, 12 of the 18 PADs could combine with thrombin and show better results than those obtained with fibrinogen. However, in AutoDock (Table 7), the averages of PADs with the two proteins were very close. AutoDock predicted that Cd. **12** had the lowest binding energy with thrombin, but in fact Cd. **13** had the smallest *K*_D_, indicating that this compound had the best combination with thrombin.

For *p*-hydroxycinnamic acid derivatives, AutoDock predicted that Cds. **9**–**12** would have a better combination with thrombin when the side chain was longer. Indeed, as shown in Table 8, Cd. **9** had a smaller *K*_D_ due to its shorter side chain. The reason for this finding was that when the side chain got increasingly longer, the hydrophobicity of the molecule increased, and because the conditions of the assays and the environment in actual human bodies involved water as the major solvent, increasing hydrophobicity would decrease the interactions between small molecules and proteins. This phenomenon was very obvious in the SPR results for Cd. **13**. Since the structure of Cd. **13** had two additional hydroxyl groups, which could decrease the hydrophobicity and increase its hydrophilicity, Cd. **13** would have better combination results with proteins under the assay conditions (water was the major solvent).

In AutoDock, *p*-hydroxycinnamic acid derivatives had the lowest average binding energy with fibrinogen compared with the other two derivatives. In SPR, although only two *p*-hydroxycinnamic acid derivatives had *K*_D_ values, *p*-hydroxycinnamic acid derivatives remained the best among the three PADs series.

Although SPR provided a realistic combination of small molecules and proteins, the results did not show the effects on protein activities. To examine the changes (enhance or inhibit activity) of protein activities after combination, plasma recalcification time (PRT), PT, APTT, FIB, and thrombin time (TT) were examined.

### 3.4. PRT, PT, APTT, FIB, and TT Results

Five blood assays were used to determine the procoagulant and anticoagulation activities of 18 PADs, and the results are listed in Table 9.

Cds. **8** and **13** had the best combination results with thrombin in SPR and showed significant anticoagulation activities in all five assays, which might indicate that these compounds could inhibit thrombin activity in combination and make the conversion from soluble fibrinogen to insoluble fibrin without thrombin promotion. Cd. **16** was the second compound in the 18 PADs, which had significant anticoagulation activity in all five blood assays. However, this compound did not combine with thrombin and showed weak combination with fibrinogen (large *K*_D_ value). These results showed that the anticoagulation activity of Cd. **16** might be caused by the combination with other proteins or factors in the coagulation (clotting) cascade (See Figure 1). Cd. **9** showed combined results with thrombin and fibrinogen in SPR, but the results with fibrinogen had a large *K*_D_ value, indicating that the combination was extremely weak. No obvious effect in the FIB assay confirmed that Cd. **9** had little effect on fibrinogen, and its anticoagulation activity might be caused by the combination with thrombin.

Although Cds. **8**, **9**, **13**, and **16** all had the better anticoagulation activities among the 18 PADs, these compounds belonged to two different phenolic acid derivatives: Cds. **8**, **9**, and **13** were members of the *p*-hydroxycinnamic acid series and Cd. **16** was a member of the ferulic acid series, indicating that the anticoagulation activities of these compounds affected different proteins or factors in the coagulation cascade. Even in the same phenolic acid derivative, Cds. **8** and **13**, showing the best anticoagulation activity, had different SPR results.

Both Cds. **16** and **5** had good anticoagulation activity, and the former was the best among the 18 PADs (See Table 9). This activity difference was triggered by the only difference in their structures, the 3-position of the benzene ring. The hydroxyl group in the 3-position of the benzene ring was changed to a methoxy group. This change made Cd. **16** bigger and much easier to fit the receptor (proteins or factors in the coagulation cascade).

Cd. **4** had the best procoagulant activity among the 18 PADs. However, the combination (see Table 8) with thrombin was very weak, and no combination was observed with fibrinogen. Cds. **6** and **7** had similar results compared to Cd. **4**. These phenomena indicated that the procoagulant activities of Cds. **4**, **6**, and **7** were achieved via combining with other proteins or factors in the coagulation cascade rather than with thrombin and fibrinogen. This finding might be due to their structures. All three compounds had very long R groups in their structures, and the distances between the acylamino N and the carbonyl non-hydrogen atoms were very close (see Table 4 and the Supplementary Information). Cd. **4** showed the best procoagulant activity among the 18 PADs, indicating that its R structure was suitable to combine with some proteins or factors in the coagulation cascade and subsequently promote procoagulation. However, the three key distances of Cds. **6** and **7** were shorter than those of Cd. **4**, particularly N–hydroxyl O of −COOH and N–C of −COOH, suggesting that the combinations between Cds. **6** or **7** and the proteins or factors were less efficient compared with the combination between Cd. **4** and the proteins or factors.

Mackman [50] classified anticoagulants into three main types: Vitamin K antagonists, heparins, and direct inhibitors of factor Xa and thrombin. For the first type, Vitamin K antagonists function by inhibiting the enzyme vitamin K epoxide reductase, which uses vitamin K to post-translationally modify several coagulation proteins (factor VII, factor IX, factor X and prothrombin). The second type, heparin, binds to the protein antithrombin and markedly increases the ability of this protein to inhibit factor Xa and thrombin. The third type has two typical examples, lepirudin and desirudin. However, Correia-da-Silva and co-workers [51] demonstrated that some persulfated small molecules might be a fourth type of anticoagulant, with dual anticoagulant and antiplatelet effects. The structures of the persulfated compounds in [50] and the PADs synthesized in the present study showed some similarities, such as benzene rings, phenolic hydroxyl groups, carbonyl groups, and sulfo groups. This evidence suggests that the PADs might have similar functions with dual effects.

## 4. Conclusions

According to the results, particularly the data from the five blood assays, the seven caffeic acid derivatives all had certain anticoagulant or procoagulant activities; in the *p*-hydroxycinnamic acid series, two derivatives, Cds. **8** and **13**, had the best anticoagulant activities among all 18 PADs. Among the ferulic acid derivatives, one compound (Cd. **16**) showed the best anticoagulant activity but combined with a protein or factor that differed from the target of Cds. **8** and **13**.

The anticoagulant or procoagulant activities of PADs slightly changed after structure modification. Considering the complexities of haemostasis in vivo and the process of promoting blood circulation for removing blood stasis, the method used in the present study provided some important data for molecule design. In the future, the method (based on the SPR to obtain the combination data between proteins or factors in the coagulation pathway and synthesized compounds and was assisted by Materials Studio software to optimize the structures of the synthesized compounds and calculate the significant structure information, such as cloud density) will be continually used to provide additional information and data for designing molecules with in vivo activities of haemostasis and promoting blood circulation for removing blood stasis.

## Figures and Tables

**Table 1:** The yields, colours, and ESI-MS data of Cds. **1**–**7**.

	Cd. 1	Cd. 2	Cd. 3	Cd. 4	Cd. 5	Cd. 6	Cd. 7
Yield (%)	53.2	48.3	45.1	65.3	41.3	66.3	69.6
Color	White powder	White powder	Yellow needle crystal	White needle crystal	Yellow powder	While needle crystal	Light yellow powder
m.p. (°C)	>244 ^a^	180–181	229–230	164–165	>200 ^a^	162–163	178–180
ESI-MS (*m*/*z*)	238.05 ^b^	250.08 ^c^	264.07 ^c^	292.3 ^c^	286.2 ^c^	318.3 ^c^	312.3 ^c^

^a^ Decomposing temperature; ^b^ [M + 1]^+^; ^c^ [M − 1]^−^.

**Table 2:** The IR data of Cds. **1**–**7** (KBr pellets, ν_max_, cm^−1^).

	Cd. 1	Cd. 2	Cd. 3	Cd. 4	Cd. 5	Cd. 6	Cd. 7
*ν*_−OH(COOH)_	3537.51, 3248.05	3629.55, 3350.69	3650.56, 3136.97	3496.45, 3101.13	3181.75	3495.45	3118.15
*ν*_−NH-_	3379.64	3409.00	3363.27	3307.25	3411.44	3346.95	3274.45
*ν*_−CO−_	1646.80	1655.01	1647.46	1649.79	1704.00	1715.59	1689.94
*ν*_>C=C<_	1622.51	1624.10	1618.62	1598.80	1654.61	1642.14	1650.91
*ν*_Ar_	1577.87, 1458.28	1598.39, 1456.53	1618.62, 1454.79	1567.55, 1520.48	1592.87, 1454.86	1598.02, 1460.39	1608.57, 1531.97
*ν*_C−O(ArOH)_	1263.63	1277.08	1304.95	1272.29	1309.42	1299.67	1268.80

**Table 3:** The yields, colours, and ESI-MS data of Cds. **8**–**14**.

	Cd. 8	Cd. 9	Cd. 10	Cd. 11	Cd.12	Cd. 13	Cd. 14
Yield (%)	45.1	61.2	70.2	86.3	85.3	85.5	93.5
Color	White powder	White powder	White powder	Light yellow lamellar crystal	Orange lamellar crystal	White powder	Colorless needle crystal
m.p. (°C)	>300 ^a^	>246 ^a^	172–173	237–238	209–210	288–289	231–232
ESI-MS (*m*/*z*)	243.00 ^c^	220.1 ^c^	250.18 ^b^	278.3 ^b^	306.3 ^b^	282.2 ^b^	262.2 ^c^

^a^ Decomposing temperature; ^b^ [M + 1]^+^; ^c^ [M − 1]^−^.

**Table 4:** The IR data of Cds. **8**–**14** (KBr pellets, ν_max_, cm^−1^).

	Cd. 8	Cd. 9	Cd. 10	Cd. 11	Cd.12	Cd. 13	Cd. 14
*ν*_−OH(COOH)_	3595.95, 3230.77	3573.49, 3161.69	3573.23, 3463.56	3573.06, 3203.06	3573.53, 3158.49	3521.79, 3111.08	3574.05, 3202.35
*ν*_−CO−_	1701.16	1654.02	1645.38	1700.59	1702.40	1647.02	1722.54
*ν*_>C=C<_	1634.48	1608.00	1645.38	1635.38	1637.53	1620.00	1644.43
*ν*_Ar_	1591.88, 1517.66	1559.81, 1473.85	1603.91, 1518.98	1581.23, 1521.24	1581.44, 1520.35	1580.19, 1525.42	1578.57, 1514.76
*ν*_C−O(ArOH)_	1271.59	1268.99	1294.21	1262.57	1299.67	1299.93	1291.03

**Table 5:** The yields, colours, and ESI-MS data of Cds. **15**–**18**.

	Cd. 15	Cd. 16	Cd. 17	Cd. 18
Yield (%)	84.1	85.7	85.5	87.3
Color	White powder	Yellow needle crystal	White powder	White powder
m.p. (°C)	61–62	285–286	188–189	228–229
ESI-MS (*m*/*z*)	306.3 ^b^	300.62 ^b^	334.4 ^a^	326.2 ^b^

^a^ [M + 1]^+^; ^b^ [M − 1]^−^.

**Table 6:** The IR data of Cds. **15**–**18** (KBr pellets, ν_max_, cm^−1^).

	Cd. 15	Cd. 16	Cd. 17	Cd. 18
*ν*_−OH(COOH)_	3522.27	3626.78, 3076.53	3370.63	3464.64
*ν*_−NH−_	3311.60	3313.74	3444.83	3282.88
*ν*_−CO−_	1693.84	1650.47	1704.85	1688.55
*ν*_>C=C<_	1650.48	1617.38	1655.58	1649.58
*ν*_Ar_	1583.13, 1469.73	1597.79, 1516.69	1607.74, 1514.88	1608.13, 1514.88
*ν*_−SO−_	N/A	1345.22, 1155.91	N/A	N/A
*ν*_C−O(ArOH)_	1299.40	1287.63	1288.64	1278.45

**Table 7:** The binding energies (kJ∙mol^−1^) of the 18 phenolic acid derivatives with seven protein targets.

No.	3RLW	1YPK	2J4I	3GHG	2ANT	1QRZ	3H5C
Cd. **1**	−22.50	−16.81	−16.02	−15.06	−17.25	−15.50	−13.13
Cd. **2**	−20.31	−17.26	−16.83	−16.23	−18.03	−16.18	−14.56
Cd. **3**	−20.60	−19.07	−17.41	−18.31	−19.92	−18.81	−15.54
Cd. **4**	−18.26	−20.75	−19.07	−19.25	−23.17	−18.31	−17.48
Cd. **5**	−18.50	−16.36	−16.48	−17.47	−17.74	−15.39	−13.44
Cd. **6**	−16.43	−22.06	−18.85	−20.40	−22.44	−19.83	−18.89
Cd. **7**	−22.75	−19.92	−18.40	−15.99	−21.11	−17.17	−17.17
Cd. **8**	−12.92	−13.87	−14.13	−15.60	−15.41	−13.58	−11.24
Cd. **9**	−18.18	−18.22	−17.20	−25.48	−20.47	−17.43	−13.69
Cd. **10**	−16.03	−21.16	−18.83	−20.94	−22.51	−18.40	−16.92
Cd. **11**	−22.11	−22.18	−19.33	−20.94	−23.08	−20.19	−19.79
Cd. **12**	−24.47	−24.77	−21.79	−23.60	−25.98	−20.29	−20.61
Cd. **13**	−19.12	−20.45	−19.28	−23.60	−21.07	−18.31	−16.44
Cd. **14**	−19.62	−20.11	−18.40	−20.58	−21.17	−18.05	−16.58
Cd. **15**	−21.47	−20.68	−19.66	−18.84	−22.91	−20.39	−19.87
Cd. **16**	−14.65	−18.73	−16.56	−17.05	−19.23	−16.57	−15.34
Cd. **17**	−20.52	−20.87	−19.87	−18.58	−23.73	−20.41	−19.68
Cd. **18**	−21.03	−21.79	−21.73	−16.37	−21.31	−17.05	−18.88
Ave.	−19.42	−19.73	−18.32	−19.13	−20.92	−17.88	−16.63

**Table 8:** The SPR results of phenolic acid derivatives with thrombin and fibrinogen.

No.	Thrombin	Fibrinogen
Cd. **1**	4.41 × 10^−6^	N/A
Cd. **2**	N/A	N/A
Cd. **3**	N/A	N/A
Cd. **4**	11.3 × 10^−6^	N/A
Cd. **5**	N/A	N/A
Cd. **6**	6.29 × 10^−6^	N/A
Cd. **7**	N/A	N/A
Cd. **8**	12.1 × 10^−6^	N/A
Cd. **9**	4.54× 10^−6^	10.1 × 10^−6^
Cd. **10**	44.7 × 10^−6^	14.5 × 10^−6^
Cd. **11**	N/A	N/A
Cd. **12**	N/A	N/A
Cd. **13**	1.70 × 10^−6^	N/A
Cd. **14**	12.1 × 10^−6^	N/A
Cd. **15**	N/A	N/A
Cd. **16**	N/A	16.2 × 10^−6^
Cd. **17**	43.5 × 10^−6^	N/A
Cd. **18**	7.77 × 10^−6^	N/A

**Table 9:** Plasma recalcification, prothrombin, activated partial thromboplastin, fibrinogen, and thrombin time results of the phenolic acid derivatives.

No.	PRT	APTT	PT	FIB	TT
Cd. **1**	Anti. at High Concen.	Anti. at High Concen.	NOE	NOE	NOE
Cd. **2**	Anti. at High Concen.	Anti. at High Concen.	NOE	NOE	NOE
Cd. **3**	NOE	NOE	Anti. at Some Concen.	NOE	Anti. at Some Concen.
Cd. **4**	Sig. Procoa.	Sig. Procoa.	Sig. Procoa.	Procoa. at Some Concen.	Procoa. at Some Concen.
Cd. **5**	Sig. Anti.	Sig. Anti.	Sig. Anti.	Anti. at Some Concen.	Anti. at Some Concen.
Cd. **6**	Sig. Procoa.	Sig. Procoa.	Sig. Procoa.	NOE	NOE
Cd. **7**	Sig. Procoa.	Sig. Procoa.	Sig. Procoa.	NOE	NOE
Cd. **8**	Sig. Anti.	Sig. Anti.	Sig. Anti.	Sig. Anti.	Sig. Anti.
Cd. **9**	Sig. Anti.	Sig. Anti.	Anti. at High Concen.	NOE	Sig. Anti.
Cd. **10**	Procoa. at High Concen.	Procoa. at High Concen.	NOE	NOE	Procoa. at Some Concen.
Cd. **11**	Sig. Procoa.	Procoa. at High Concen.	NOE	NOE	NOE
Cd. **12**	Procoa. at High Concen.	Sig. Procoa.	Procoa. at High Concen.	Procoa. at High Concen.	NOE
Cd. **13**	Sig. Anti.	Sig. Anti.	Sig. Anti.	Sig. Anti.	Sig. Anti.
Cd. **14**	Sig. Anti.	Sig. Anti.	Anti. at High Concen.	NOE	NOE
Cd. **15**	Procoa. at High Concen.	NOE	Procoa. at Some Concen.	Procoa. at Some Concen.	Procoa. at High Concen.
Cd. **16**	Sig. Anti.	Sig. Anti.	Sig. Anti.	Sig. Anti.	Sig. Anti.
Cd. **17**	Procoa. at High Concen.	Procoa. at Some Concen.	Procoa. at Some Concen.	NOE	NOE
Cd. **18**	Procoa. at High Concen.	Procoa. at High Concen.	Procoa. at Some Concen.	NOE	NOE

NOE: No obvious effect; Sig. Anti: Significant anticoagulation; Sig. Procoa: Significant procoagulant action; Anti. at High Concen.: Anticoagulation at a high concentration; Procoa. at High Concen.: Procoagulant action at a high concentration; Anti. at Some Concen.: Anticoagulation at some concentrations; Procoa. at Some Concen.: Procoagulant action at some concentrations.

## Supplementary Materials

Supplementary materials are available online.
