# Supplementary material for: Study on the Anticoagulant or Procoagulant Activities of Type II Phenolic Acid Derivatives
Source: Molecules. 2017 Nov 28;22(12):2047. doi: 10.3390/molecules22122047 (PMC6149684; doi:10.3390/molecules22122047)
Supplement: Supplementary file 1 [file molecules-22-02047-s001.pdf]

# Study on the Anticoagulant or Procoagulant Activities of Type II Phenolic Acid Derivatives

**Xuan Luo[1,2], Chuanrong Du[1], Hui Cheng[1], Jian-hua Chen[3], and Cuiwu Lin*[1,2]**

[1] School of Chemistry and Chemical Engineering, Guangxi University, 100 Daxue Road, Nanning, Guangxi, 530004, P. R. China; luo-xuan625@hotmail.com (X.L.); 408082854@qq.com (C.D.); 240514119@qq.com (H.C.)

[2] Guangxi Colleges and Universities Key Laboratory of Applied Chemistry Technology and Resource Development, Guangxi University, 100 Daxue Road, Nanning, Guangxi, 530004, P. R. China

[3] School of Resources, Environment, and Materials, Guangxi University, 100 Daxue Road, Nanning, Guangxi, 530004, P.R. China; jhchen@gxu.edu.cn (J.-H.C.)

* Correspondence: cuiwulin@163.com; Tel: +86-771-323-3718

## 1. Melting points and spectral data

### *1.1 Caffeic acid derivatives*

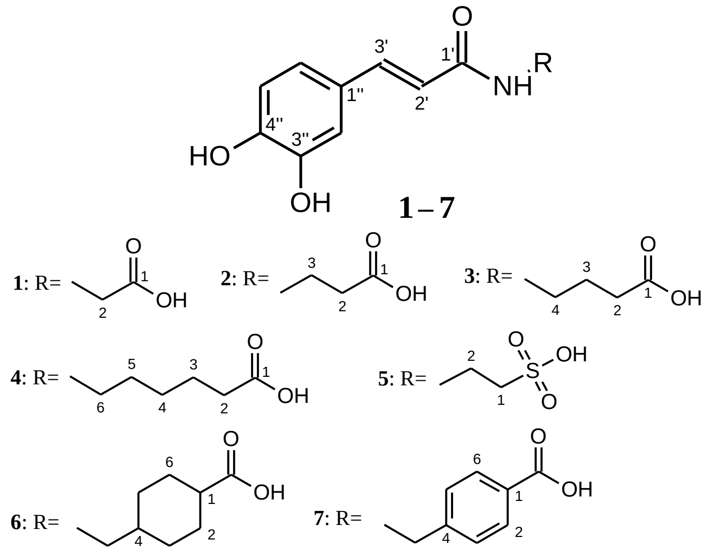


Figure S1 The Structures of Cds. **1**–**7**

Table S1 $^{13}C$ NMR Data of Cds. **1**–**7** (151 MHz, DMSO-$d_6$)

| | Cd. **1** | Cd. **2** | Cd. **3** | Cd. **4** | Cd. **5** | Cd. **6** | Cd. **7** |
|---|---|---|---|---|---|---|---|
| –COOH | N/A | N/A | N/A | N/A | N/A | 176.82 | 168.29 |
| C-1 | 172.87 | 173.43 | 177.65 | 174.59 | 50.71 | 44.80 | 131.84 |
| C-2 | 43.89 | 34.43 | 35.13 | 33.70 | 35.71 | 29.56 | 129.47 |
| C-3 | N/A | 35.41 | 25.98 | 24.34 | N/A | 28.35 | 127.13 |
| C-4 | N/A | N/A | 35.29 | 26.14 | N/A | 37.20 | 143.74 |
| C-5 | N/A | N/A | N/A | 29.06 | N/A | 28.35 | 127.13 |
| C-6 | N/A | N/A | N/A | 38.58 | N/A | 29.56 | 129.47 |
| C-1′ | 165.12 | 165.99 | 165.63 | 165.41 | 165.25 | 165.47 | 165.80 |
| C-2′ | 118.87 | 118.80 | 118.54 | 118.67 | 118.52 | 118.66 | 118.10 |
| C-3′ | 138.98 | 139.65 | 139.22 | 139.01 | 139.16 | 139.02 | 139.90 |
| C-1″ | 126.38 | 126.83 | 125.84 | 126.51 | 126.35 | 126.50 | 126.32 |
| C-2″ | 114.25 | 114.28 | 114.29 | 113.88 | 113.93 | 113.87 | 114.25 |
| C-3″ | 146.10 | 145.96 | 145.56 | 145.59 | 145.55 | 145.55 | 145.86 |
| C-4″ | 147.94 | 147.74 | 148.81 | 147.67 | 147.36 | 147.26 | 147.82 |
| C-6″ | 120.38 | 120.89 | 120.31 | 120.44 | 120.40 | 120.37 | 120.63 |
| –$CH_2$– | N/A | N/A | N/A | N/A | N/A | 42.57 | 42.16 |

Table S2 $^{1}$H NMR Data of Cds. **1**–**7** (600 MHz, DMSO-$d_6$)

| | Cd. **1** | Cd. **2** | Cd. **3** | Cd. **4** | Cd. **5** | Cd. **6** | Cd. **7** |
|---|---|---|---|---|---|---|---|
| −COOH | N/A | 12.15 (1 H, s) | N/A | 12.02 (1 H, s) | N/A | N/A | N/A |
| −OH | N/A | 9.28 (2 H, s) | N/A | 9.27 (2 H, d) | 9.24 (2 H, d) | 9.25 (2 H, d) | 4.30 (1 H, s, −OH-3″), 4.29(1 H, s, −OH-4″) |
| −CONH− | 7.57 (1 H, t, *J* = 4.80 Hz) | 8.07 (1 H, t, *J* = 5.46 Hz) | 8.17 (1 H, s) | 7.98 (1 H, t, *J* = 5.64 Hz) | 7.93 (1 H, t, *J* = 5.55 Hz) | 7.95 (1 H, t, *J* = 5.82 Hz) | 8.64 (1 H, t, *J* = 5.94 Hz) |
| H-3′ | 7.18 (1 H, d, *J* = 15.60 Hz) | 7.23 (1 H, d, *J* = 15.66 Hz) | 7.20 (1 H, d, *J* = 15.60 Hz) | 7.23 (1 H, d, *J* = 15.66 Hz) | 7.21 (1 H, d, *J* = 15.60 Hz) | 7.22 (1 H, d, *J* = 15.60 Hz) | 7.30 (1 H, d, *J* = 15.66 Hz) |
| H-2″ | 7.06 (1 H, d, *J* = 1.92 Hz | 6.94 (1 H, d *J* = 2.04 Hz) | 6.94 (1 H, s) | 6.94 (1 H, d, *J* = 1.95 Hz) | 6.94 (1 H, d, *J* = 2.04 Hz) | 6.93 (1 H, d, *J* = 1.86 Hz) | 6.99 (1 H, d, *J* = 1.92 Hz) |
| H-6″ | 6.80 (1 H, dd, *J* = 8.10, 1.92 Hz) | 6.83 (1 H, dd, *J* = 8.10, 2.04 Hz) | 6.69 (1 H, d, *J* = 7.92 Hz) | 6.83 (1 H, dd, *J* = 8.10, 1.95 Hz) | 6.84 (1 H, dd, *J* = 8.10, 2.04 Hz) | 6.83 (1 H, dd, *J* = 8.10, 1.86 Hz) | 6.85 (1 H, dd, *J* = 8.22, 2.04 H) |
| H-5″ | 6.72 (1 H, d, *J* = 8.10, Hz) | 6.74 (1 H, d, *J* = 8.10 Hz) | 6.77 (1 H, d, *J* = 7.92 Hz) | 6.74 (1 H, d, *J* = 8.10 Hz) | 6.73 (1 H, d, *J* = 8.10 H) | 6.74 (1 H, d, *J* = 8.10 H) | 6.76 (1 H, d, *J* = 8.22 Hz) |
| H-2′ | 6.55 (1 H, d, *J* = 15.60 Hz) | 6.34 (1 H, d, *J* = 15.66 Hz) | 6.30 (1 H, d, *J* = 15.60 Hz) | 6.32 (1 H, d, *J* = 15.66 Hz) | 6.28 (1 H, d, *J* = 15.60 Hz) | 6.35 (1 H, d, *J* = 15.60 Hz) | 6.43 (1 H, d, *J* = 15.66 Hz) |
| H-1 | N/A | N/A | N/A | N/A | 2.60 (2 H, t, *J* = 7.32 Hz) | 2.14–2.10 (1 H, m) | N/A |
| H-2 | 3.55 (2 H, d, *J* = 4.80 Hz) | 2.43 (2 H, t, *J* = 6.75 Hz) | 2.04 (2 H, t, *J* = 6.78) | 2.20 (2 H, t, *J* = 7.30 Hz) | 3.42 (2 H, m) | 1.90–1.73 (4 H, m) | 7.89 (2 H, d, *J* = 8.22 Hz) |
| H-3 | N/A | 3.35 (2 H, m) | 1.65 (2 H, m) | 1.52–1.39 (4 H, m) | N/A | 1.29–0.89 (4 H, m) | 7.34 (2 H, d, *J* = 8.22 Hz) |
| H-4 | N/A | N/A | 3.13 (2 H, s) | 1.32–1.25 (2 H, m) | N/A | 1.43–1.33 (1 H, m) | N/A |
| H-5 | N/A | N/A | N/A | 1.52–1.39 (4 H, m) | N/A | 1.29–0.89 (4 H, m) | 7.34 (2 H, d, *J* = 8.22 Hz) |
| H-6 | N/A | N/A | N/A | 3.14 (2 H, m) | N/A | 1.90–1.73 (4 H, m) | 7.89 (2 H, d, *J* = 8.22 Hz) |
| −$CH_2$− | N/A | N/A | N/A | N/A | N/A | 3.01 (2 H, t, *J* = 5.82 Hz | 4.43 (2 H, d, *J* = 5.94 Hz) |

*1.2 p-Hydroxycinnamic acid derivatives*

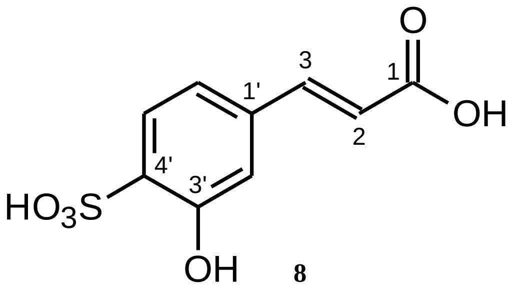


**8**

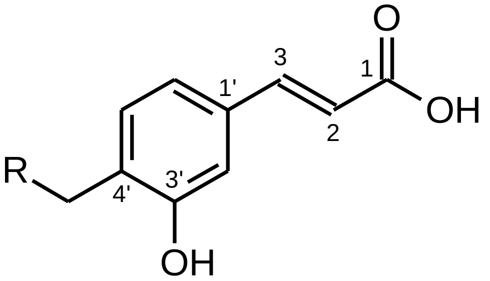


**9**−**14**

**9**: R= -N($CH_3$)$_2$ **10**: R'= -N($CH_2CH_3$)$_2$ **11**: R= -N($CH_2CH_2CH_3$)$_2$

**12**: R= -N($CH_2CH_2CH_2CH_3$)$_2$ **13**: R= -N($CH_2CH_2OH$)$_2$ **14**: R= 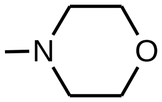


Figure S2 The Structures of Cds. **8**–**14**

Table S3 $^{13}$C NMR Data of Cds. **8**–**14** (151 MHz, DMSO-$d_6$)

| | Cd. **8** | Cd. **9** | Cd. **10** | Cd. **11** | Cd.**12** | Cd. **13** | Cd. **14** |
|---|---|---|---|---|---|---|---|
| –$CH_2$– | N/A | 57.71 | 54.95 | 53.55 | 51.66 | 54.94 | 53.41 |
| C-1 | 167.55 | 172.19 | 167.84 | 167.28 | 167.31 | 172.10 | 167.33 |
| C-2 | 118.49 | 119.76 | 118.95 | 119.53 | 119.05 | 119.80 | 118.98 |
| C-3 | 143.22 | 146.52 | 143.59 | 143.29 | 143.27 | 146.45 | 143.31 |
| C-1′ | 136.82 | 138.59 | 134.40 | 136.63 | 136.62 | 138.59 | 136.83 |
| C-2′ | 116.11 | 115.97 | 114.45 | 114.73 | 114.76 | 115.79 | 114.84 |
| C-3′ | 153.57 | 157.40 | 157.97 | 156.99 | 157.02 | 157.19 | 157.16 |
| C-4′ | 132.17 | 119.72 | 125.15 | 118.68 | 118.60 | 119.50 | 117.57 |
| C-5′ | 127.67 | 133.86 | 129.21 | 133.47 | 133.53 | 133.71 | 133.98 |
| C-6′ | 120.27 | 121.41 | 119.17 | 120.12 | 120.08 | 121.64 | 120.14 |
| C-1″ | N/A | 43.49 | 45.90 | 50.49 | 50.20 | 56.21 | 63.05 |
| C-2″ | N/A | N/A | 10.98 | 16.53 | 24.82 | 55.95 | 50.72 |
| C-3″ | N/A | N/A | N/A | 11.00 | 19.53 | N/A | N/A |
| C-4″ | N/A | N/A | N/A | N/A | 13.53 | N/A | N/A |

Table S4 $^{1}$H NMR Data of Cds. **8**–**14** (600 MHz, DMSO-$d_6$)

| | Cd. **8** | Cd. **9** | Cd. **10** | Cd. **11** | Cd.**12** | Cd. **13** | Cd. **14** |
|---|---|---|---|---|---|---|---|
| −COOH | N/A | N/A | N/A | 10.65 (1 H, s) | 12.49 (1 H, s) | 11.29 (1 H, s) | 12.50 (1 H, s) |
| −OH | 10.52 (1 H, s) | N/A | N/A | 10.01 (1 H, s) | 10.18 (1 H, s) | N/A | 10.64 (1 H, s) |
| $R_3NH^+$ | N/A | N/A | N/A | N/A | 10.71 (1 H, s) | N/A | 11.05 (1 H, s) |
| $-CH_2-$ | N/A | 4.37 (2 H, s) | 3.75 (2 H, s) | 4.21 (2 H, s) | 4.22 (2 H, s) | 4.59 (2 H, s) | 4.25 (2 H, s) |
| H-2 | 6.48 (2 H, d, *J* = 16.02 Hz) | 6.52 (1 H, d, *J* = 15.96 Hz) | 6.97 (1 H, s) | 6.44 (1 H, d, *J* = 15.96 Hz) | 6.44 (1 H, d, *J* = 15.96 Hz) | 6.53 (1 H, d, *J* = 15.96 Hz) | 6.44 (1 H, d, *J* = 15.96 Hz) |
| H-3 | 7.49 (1 H, d, *J* = 16.02 Hz) | 7.70 (1 H, d, *J* = 15.96 Hz) | 7.46 (1 H, d, *J* = 15.96 Hz) | 7.49 (1 H, d, *J* = 15.96 Hz) | 7.49 (1 H, d, *J* = 15.96 Hz) | 7.70 (1 H, d, *J* = 15.96 Hz) | 7.50 (1H, d, *J* = 15.96 Hz) |
| H-2′ | 7.09 (1 H, d, *J* = 1.68 Hz) | 7.25 (1 H, d, *J* = 1.08 Hz) | 6.42 (1 H, d, *J* = 15.96 Hz) | 7.24 (1 H, s) | 7.26 (1 H, s) | 7.26 (1 H, s) | 7.24 (1 H, d, *J* = 1.26 Hz) |
| H-5′ | 7.46 (1 H, d, *J* = 7.86 Hz) | 7.40 (1 H, d, *J* = 7.80, 1.08 Hz) | 7.03 (1 H, d, *J* = 7.80 Hz) | 7.19 (1 H, d, *J* = 7.86 Hz) | 7.58 (1 H, d, *J* = 7.92 Hz) | 7.18 (1 H, d, *J* = 7.86 Hz) | 7.58 (1 H, d, *J* = 7.86 Hz) |
| H-6′ | 7.13 (1 H, dd, *J* = 7.86, 1.68 Hz) | 7.18 (1 H, d, *J* = 7.80 Hz) | 7.10 (1 H, d, *J* = 7.80 Hz) | 7.54 (1 H, d, *J* = 7.86 Hz) | 7.19 (1 H, d, *J* = 7.92 Hz) | 7.40 (1 H, d, *J* = 7.86 Hz) | 7.20 (1 H, dd, *J* = 7.86, 1.26 Hz) |
| H-1″ | N/A | 2.93 (6 H, s) | 2.56 (4 H, m) | 2.93 (4 H, t, *J* = 8.04 Hz) | 2.96 (4 H, t) | 3.50 (4 H, t, *J* = 4.92 Hz) | 3.86 (4 H, m) |
| H-2″ | N/A | N/A | 1.02 (6 H, t, *J* = 7.14 Hz) | 1.73 (4H, m, H-2″) | 1.73–1.68 (4 H, m) | 4.05 (4 H, t, *J* = 4.92 Hz | 3.16 (4 H, m) |
| H-3″ | N/A | N/A | N/A | 0.86 (6H, t, *J* = 7.32 Hz) | 1.28–1.25 (4 H, m) | N/A | N/A |
| H-4″ | N/A | N/A | N/A | N/A | 0.88–0.85 (6H, t, *J* = 7.38 Hz) | N/A | N/A |

*1.3 Ferulic acid derivatives*

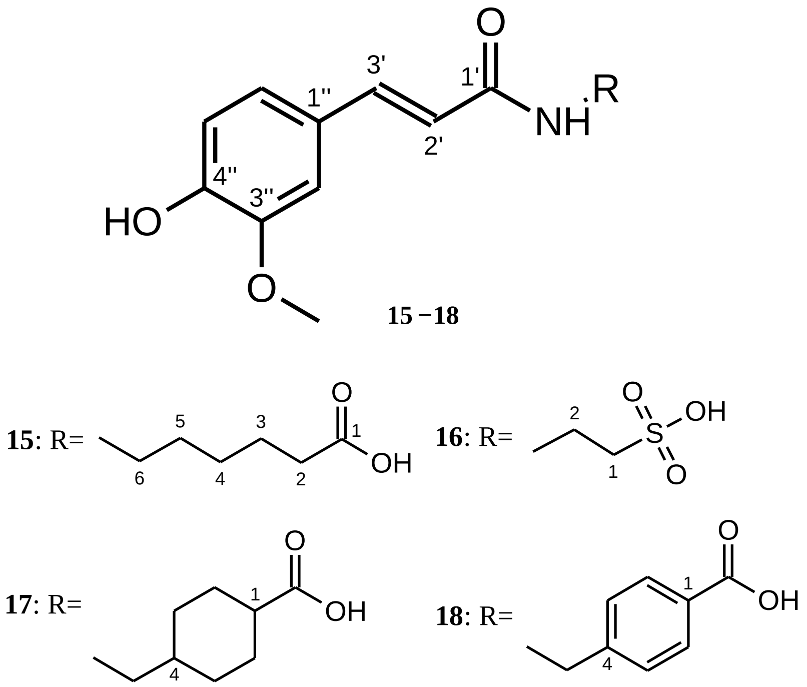


Figure S3 The Structures of Cds. **15**–**18**

Table S5 $^{13}C$ NMR Data of Cds. **15**–**18** (151 MHz, DMSO-$d_6$)

| | Cd. **15** | Cd. **16** | Cd. **17** | Cd. **18** |
|---|---|---|---|---|
| –COOH | N/A | N/A | 176.77 | 167.23 |
| –$OCH_3$ | 55.98 | 58.54 | 55.51 | 55.55 |
| $CH_2$– | N/A | N/A | 42.52 | 42.07 |
| C-1 | 174.49 | 52.66 | 44.75 | 126.35 |
| C-2 | 34.09 | 38.15 | 29.51 | 129.44 |
| C-3 | 24.72 | N/A | 28.31 | 127.27 |
| C-4 | 26.51 | N/A | 37.17 | N/A |
| C-5 | 29.42 | N/A | 28.31 | 127.27 |
| C-6 | 39.96 | N/A | 29.51 | 129.44 |
| C-1′ | 165.73 | 171.60 | 165.39 | 165.61 |
| C-2′ | 119.56 | 119.96 | 119.12 | 118.59 |
| C-3′ | 139.24 | 144.03 | 138.83 | 139.63 |
| C-1″ | 126.93 | 130.04 | 126.50 | 129.36 |
| C-2″ | 111.16 | 113.80 | 110.65 | 110.87 |
| C-3″ | 148.65 | 150.21 | 148.20 | 148.39 |
| C-4″ | 148.27 | 149.77 | 147.82 | 144.82 |
| C-5″ | 116.10 | 118.21 | 115.65 | 115.69 |
| C-6″ | 121.95 | 125.35 | 121.51 | 121.66 |

Table S6 $^{1}H$ NMR Data of Cds. **15**–**18** (600 MHz, DMSO-$d_6$)

| | Cd. **15** | Cd. **16** | Cd. **17** | Cd. **18** |
|---|---|---|---|---|
| –COOH | 12.00 (1 H, s) | N/A | 12.00 (1 H, s) | 12.85 (1 H, s) |
| –OH | 9.41 (1 H, s) | N/A | 9.40 (1 H, s) | 9.46 (1 H, s) |
| –CONH– | 7.93 (1 H, t, *J* = 5.49 Hz) | N/A | 7.93 (1 H, t, *J* = 6.27 Hz) | 8.56 (1 H, t, *J* = 6.00 Hz) |
| –$OCH_3$ | 3.80 (3 H, s) | 3.79 (3 H, s) | 3.80 (3 H, s) | 3.80 (3 H, s) |
| –$CH_2$– | N/A | | 3.02 (2 H, t, *J* = 6.27 Hz) | 4.46 (2 H, d, *J* = 6.00 Hz) |
| H-1 | N/A | 3.64 (2 H, t, *J* = 6.93 Hz) | 2.15–2.09 (1 H, m) | N/A |
| H-2 | 2.20 (2 H, t, *J* = 7.38 Hz) | 3.14 (2 H, t, *J* = 6.93 Hz) | 1.91–1.74 (2 H, m) | 7.91 (1 H, d, *J* = 8.28 Hz) |
| H-3 | 1.53–1.41 (2 H, m) | N/A | 1.29–0.90 (2 H, m) | 7.40 (1 H, d, *J* = 8.28 Hz) |
| H-4 | 1.31–1.26 (2 H, m) | N/A | 1.41–1.35 (1 H, m) | N/A |
| H-5 | 1.53–1.41 (2 H, m) | N/A | 1.29–0.90 (2 H, m) | 7.40 (1 H, d, *J* = 8.28 Hz) |
| H-6 | 3.14 (2 H, m) | N/A | 1.91–1.74 (2 H, m) | 7.91 (1 H, d, *J* = 8.28 Hz) |
| H-2′ | 6.43 (1 H, d, *J* = 15.66 Hz) | 6.21 (1 H, d, *J* = 15.78 Hz) | 6.47 (1 H, d, *J* = 15.72 Hz) | 6.53 (1 H, d, *J* = 15.72 Hz) |
| H-3′ | 7.30 (1 H, d, *J* = 15.66 Hz) | 7.21 (1 H, d, *J* = 15.78 Hz) | 7.31 (1 H, d, *J* = 15.72 Hz) | 7.38 (1 H, d, *J* = 15.72 Hz) |
| H-2″ | 7.11 (1 H, s) | 6.95 (1 H, t, *J* = 1.74 Hz) | 7.11 (1 H, d, *J* = 1.74 Hz) | 7.15 (1 H, d, *J* = 1.86 Hz) |
| H-5″ | 6.98 (1 H, d, *J* = 8.10 Hz) | 6.79 (1 H, t, *J* = 8.04 Hz) | 6.78 (1 H, d, *J* = 8.10 Hz) | 6.80 (1 H, d, *J* = 8.16 Hz) |
| H-6″ | 6.78 (1 H, d, *J* = 8.10 Hz) | 6.94 (1 H, dd, *J* = 8.04, 1.74 Hz) | 6.98 (1 H, dd, *J* = 8.10, 1.74 Hz) | 7.01 (1 H, dd, *J* = 8.16, 1.86 Hz) |

**2. Molecule Structures of Compounds 4, 6, and 7**

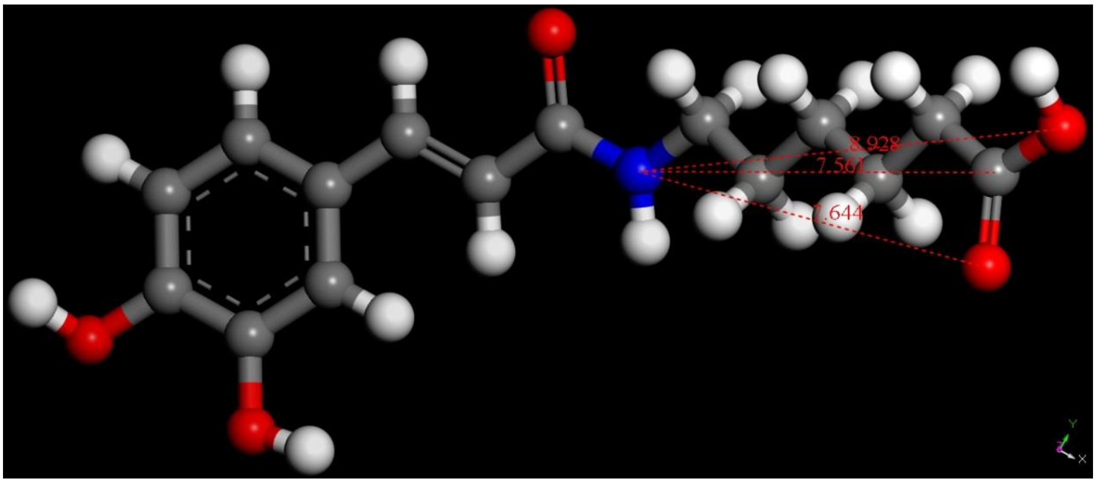


Figure 1 Molecule Structures of Cd. 4

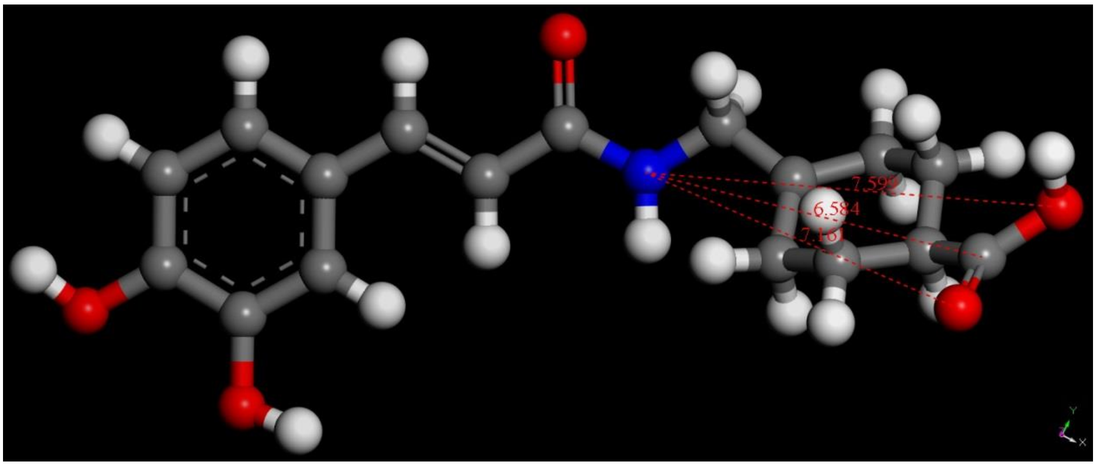


Figure 2 Molecule Structures of Cd. 6

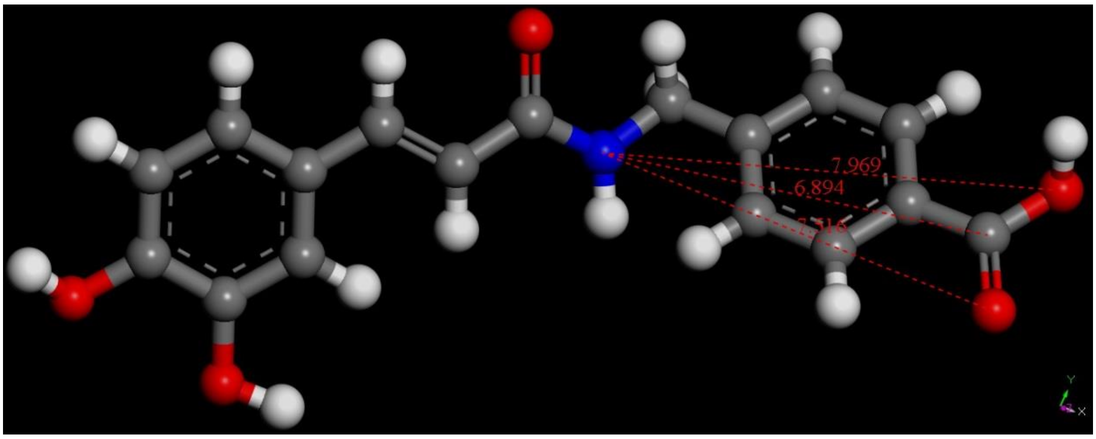


Figure 3 Molecule Structures of Cd. 7
